# Computed Tomography Features of Canine Mammary Tumours: A Retrospective Multicentre Study †

**DOI:** 10.3390/ani16213507

**Published:** 2026-11-06

**Authors:** Daniela De Felice, Stefano Spada, Luiz Paulo Nogueira Aires, Alfonso Calabria, Dario Costanza, Adelaide Greco, Rossella Terragni, Massimo Vignoli, Marco Russo

**Affiliations:** 1 Department of Veterinary Medicine and Animal Production, University of Naples “Federico II”, Via Federico Delpino, 80137 Naples, Italy; daniela.defelice2@unina.it (D.D.F.); marco.russo@unina.it (M.R.); 2 Clinic of Reproductive Medicine, Vetsuisse Faculty, University of Zurich, 8057 Zurich, Switzerland; stefano.spada@uzh.ch; 3 Interdepartmental Veterinary Radiology Center, Department of Veterinary Medicine and Animal Productions, University of Napoli “Federico II”, Via Federico Delpino 1, 80137 Naples, Italy; dario.costanza@unina.it; 4 “Calabria” Veterinary Specialty Center, Via Amerigo Vespucci, 1 Marigliano (NA), 80034 Naples, Italy; centroveterinariocalabria@gmail.com; 5 “PetCare” Veterinary Clinic, Via Marzabotto 1/2/MN, 40133 Bologna, Italy; terragni.rossella@gmail.com; 6 Department of Veterinary Medicine, University of Teramo, Nepezzano, Piano D’accio (TE), 64100 Teramo, Italy; mvignoli@unite.it

**Keywords:** canine mammary tumours, computed tomography, BI-RADS™, modified BI-RADS™, veterinary reproduction, diagnostic imaging, malignancy

## Abstract

Mammary tumours are among the most common neoplastic disorders in female dogs, and although computed tomography (CT) is widely used for oncological staging, CT evaluation of primary mammary tumours remains relatively underexplored. This multicentre retrospective study aimed to develop a standardised CT reporting protocol using a modified BI-RADS™ lexicon and to evaluate the qualitative and quantitative features associated with malignancy. Pre- and post-contrast CT scans were evaluated for 34 female dogs with 106 histologically confirmed mammary tumours, of which 81 were malignant and 25 were benign. The heterogeneous and rim enhancement patterns were strongly associated with malignant lesions (*p* < 0.001; 88.9% sensitivity, 40% specificity). Perilesional oedema exhibited high specificity (84%) but low sensitivity (33.3%). A tumour volume >1.90 cm^3^ was significantly associated with malignancy (*p* = 0.046). The proposed cumulative scoring system (0–4 points) showed an AUC of 0.711. After accounting for within-dog clustering, the dichotomous high-risk classification (score ≥ 2) remained significantly associated with malignancy (OR = 1.70; 95% CI: 1.11–2.58; *p* = 0.014). Tumours classified as high-risk (score ≥ 2) showed an 84.2% observed proportion of malignancy, with 82.7% sensitivity and 58% specificity. This CT-based protocol could help to standardise reporting and identify tumours with a higher risk of malignancy.

## Simple Summary

Mammary tumours are the most common tumours in female dogs; they can exhibit different biological characteristics, which means it is important to identify the nodules that are most probably malignant. Computed tomography is often used for tumour staging, but the tomography characteristics of tumours remain poorly described. In this retrospective study, 106 mammary tumours from 34 different female dogs were evaluated, comparing their tomographic characteristics with the histological diagnosis. The BI-RADS system, previously described in other studies for use in dogs, was also employed, adapting it to the computed tomography lexicon. Malignant tumours were associated with a higher incidence of heterogeneous and rim enhancement, the presence of perilesional oedema, and larger volumes. Based on these characteristics, a scoring system is described which demonstrates a moderate ability to identify tumours at high risk of malignancy. This tomographic description system for mammary tumours could help to standardise radiological reports and facilitate communication between radiologists, oncologists and surgeons, without replacing histopathological diagnosis.

## 1. Introduction

Mammary tumours are among the most common neoplastic disorders in women and in female dogs [1,2]. These tumours share several biological, clinical, pathological and molecular features, and constitute a significant concern both in veterinary and human public health [3]. Canine mammary tumours (CMTs) account for approximately 13.4% of all tumours diagnosed in dogs. They occur most frequently in intact females or in dogs spayed later in life, with reported prevalence rates reaching up to 41.7% in these populations [4,5,6]. Although a specific breed predisposition has not yet been identified, CMTs show a higher frequency in pure-breed dogs compared with mixed-breed dogs of similar size [7].

While the prevalence of benign and malignant mammary neoplasia varies across different epidemiological studies, malignant tumours are estimated to represent 50 to 70% of cases in bitches [8,9,10]. The occurrence of benign mammary tumours is higher in intact bitches older than 7 to 9 years old, with differences reported between large and small breeds [10]. However, it has been shown that benign mammary tumours may become malignant with time [4].

Malignant CMTs can metastasize to other organs depending on the type of tumour, localisation and histologic differentiation. In particular, malignant mammary epithelial tumours have a tendency to metastasize to regional lymph nodes and via the lymphatic system to other organs, commonly the lungs, even though they may also spread to the liver, brain and bones [11]. Conversely, malignant mesenchymal mammary tumours can metastasize haematogenously, causing direct metastasis in the lungs [3].

Although conventional staging based on the modified “TNM” staging system primarily considers tumour size [12], malignancy can also be observed in tumours smaller than 1 cm, with an incidence of 18% [7]. Therefore, early detection is imperative for a favourable outcome. For this purpose, diagnostic imaging studies are mandatory for a proper and early oncological staging.

In veterinary medicine, canine patients with mammary neoplasia undergo various imaging modalities for staging and monitoring, which primarily include thoracic radiography (XR) and abdominal and mammary ultrasonographic (US) techniques [13]. Computed tomography (CT) is an advanced imaging technique able to acquire a large number of cross-sectional images of a patient’s body, enabling multiplanar reconstructions, improving visualisation of the anatomical structures and aiding abnormalities detection [13]. The diagnostic value of XR, US and CT in the management of patients affected by CMTs differs due to their ability to detect metastasis, particularly in the lungs, consequently influencing staging and treatment decision-making [14]. Some studies have reported limitations of three-projection XR examination for the detection of pulmonary nodules, particularly in large to giant breeds [15]. In contrast, whole-body CT scans (WBCT) have been reported to increase the diagnostic yield for oncological staging in veterinary practice, and also being more precise in the detection of small lung nodules [16].

When considering direct imaging of mammary tumours, CT is not considered one of the primary methods [17]; however, thoracic, abdominal and WBCT scans include the mammary glands, and some abnormalities can potentially be identified from them [14]. In human medicine, it has already been reported that breast tumours can be incidental findings of thoracic CT studies, and that some nodules are better identified with this technique compared to mammography [18]. However, in veterinary radiology, due to the limited scientific data regarding the CT appearance of CMTs and their association with malignancy, further research is still required. These lesions may be misinterpreted or over-looked, negatively impacting patient prognosis when considering the high prevalence of malignant mammary tumours, their rapid progression, and their metastatic potential.

In human medicine, a reporting system named BI-RADS™ has been implemented to report breast imaging studies to provide a standardised lexicon across different imaging modalities, increasing repeatability, improving risk assessment and management, facilitating communication among physicians and monitoring changes over time [19]. In veterinary medicine, there is great divergency in the sonographic terminology used to describe mammary tumours, thus limiting the quality of reporting and risk assessment of bitches with mammary neoplasia [17]. A modified BI-RADS™ reporting system was proposed in a previous canine sonographic study [20], providing an adequate lexicon for veterinary radiologists to report the sonographic features of mammary tumours.

The feasibility of using CT in the assessment of CMTs was documented by Kim et al. (2017) [21], who reported a sensitivity and specificity of 83.3% for identifying malignant lesions through the analysis of calcifications. We hypothesised that specific qualitative and quantitative CT features of CMTs are associated with tumour malignancy and can be systematically characterised using an adapted canine modified BI-RADS classification system. Furthermore, we hypothesised that the application of a standardised CT reporting protocol would improve the consistency of lesion characterisation and facilitate the identification of imaging features predictive of malignancy. Therefore, this study had three objectives. First, to assess the potential of CT to identify and characterise imaging findings that may aid in differentiating benign from malignant CMTs. Second, to develop a standardised CT reporting protocol for CMTs, by evaluating CT qualitative features using an adapted canine modified BI-RADS classification system and a quantitative CT attenuation measurement to investigate their association with tumour malignancy. Third, to evaluate the utility of the modified BI-RADS system for the description and the classification of CMTs, with the aim of standardising terminology.

## 2. Materials and Methods

### 2.1. Study Design and Collection of Cases

Medical records of client-owned bitches diagnosed with CMTs who underwent WBCT study for oncological staging between January 2022 and October 2025 were retrospectively reviewed. Cases were collected from three different facilities: the Teaching Veterinary Hospital, Department of Veterinary Medicine and Animal Production, University of Naples “Federico II”; the Veterinary Hospital “Pet Care” (Bologna, Italy); and the Veterinary Hospital “Calabria” (Marigliano, Naples, Italy).

For the present study, the inclusion criteria were as follows: female canine patients of any age, breed, or weight; presence of single or multiple CMTs confirmed by histopathological examination; a surgical or biopsy procedure within a maximum of six months of CT examination, including definitive neoplastic type and grading; and complete pre- and post-contrast imaging. The exclusion criteria included lack of definitive histopathological records or patients with incomplete contrast-enhanced CT studies or patients with other mammal pathologies.

CT examinations were performed using two different multidetector CT scanners. A 16-slice scanner (BrightSpeed 16, GE Healthcare, Milwaukee, WI, USA) was used at the Teaching Veterinary Hospital, Department of Veterinary Medicine and Animal Production, University of Naples “Federico II”; at the Veterinary Hospital “Calabria” and the Veterinary Hospital “Pet Care”, a 32-slice scanner (Optima CT540, GE Healthcare, Milwaukee, WI, USA) was used.

Images were reconstructed using soft-tissue (“Standard”), bone (“Bone”), and lung (“Lung”) algorithms with a slice thickness of 1 mm and a reconstruction interval of 0.5 mm. The acquisition parameters included a pitch of 0.938:1, tube voltage of 120 kVp, gantry rotation time of 0.5 s, and adaptive tube current modulation. Post-contrast images were acquired 60–90 s following intravenous administration of iodinated contrast medium (Iopamiro 380 mg I/mL; Bracco Imaging S.p.A., Milan, Italy) at a dose of 760 mg I/kg body weight (equivalent to 2 mL/kg), followed by a saline flush using an automatic power injector (MEDRAD Vistron CT Injection System, MEDRAD Inc., Indianola, PA, USA; OptiVantage DH, Mallinckrodt Inc., Cincinnati, OH, USA; and EmpowerCTA+ Bracco Injeneering SA, Lausanne, Switzerland). The injection rate was adjusted according to patient size and ranged from 1.0 to 3.5 mL/s. Due to the retrospective nature of the study, anaesthetic protocols were not standardised for all dogs.

### 2.2. CT Images Analysis

All CT studies were reviewed in consensus by two PhD students in Animal Reproduction and Veterinary Radiology (D.D.F., L.N.P.) under the supervision of a board-certified radiologist (M.V., Dip.ECVDI). The reviewers were blinded to the medical record findings. The imaging studies were evaluated using dedicated DICOM Viewer software (RadiAnt DICOM Viewer 2025.2). For the evaluation of the CMTs, a soft-tissue window was used (WL 60, WW 400) for the both pre- and post-contrast study.

**Figure 1:**
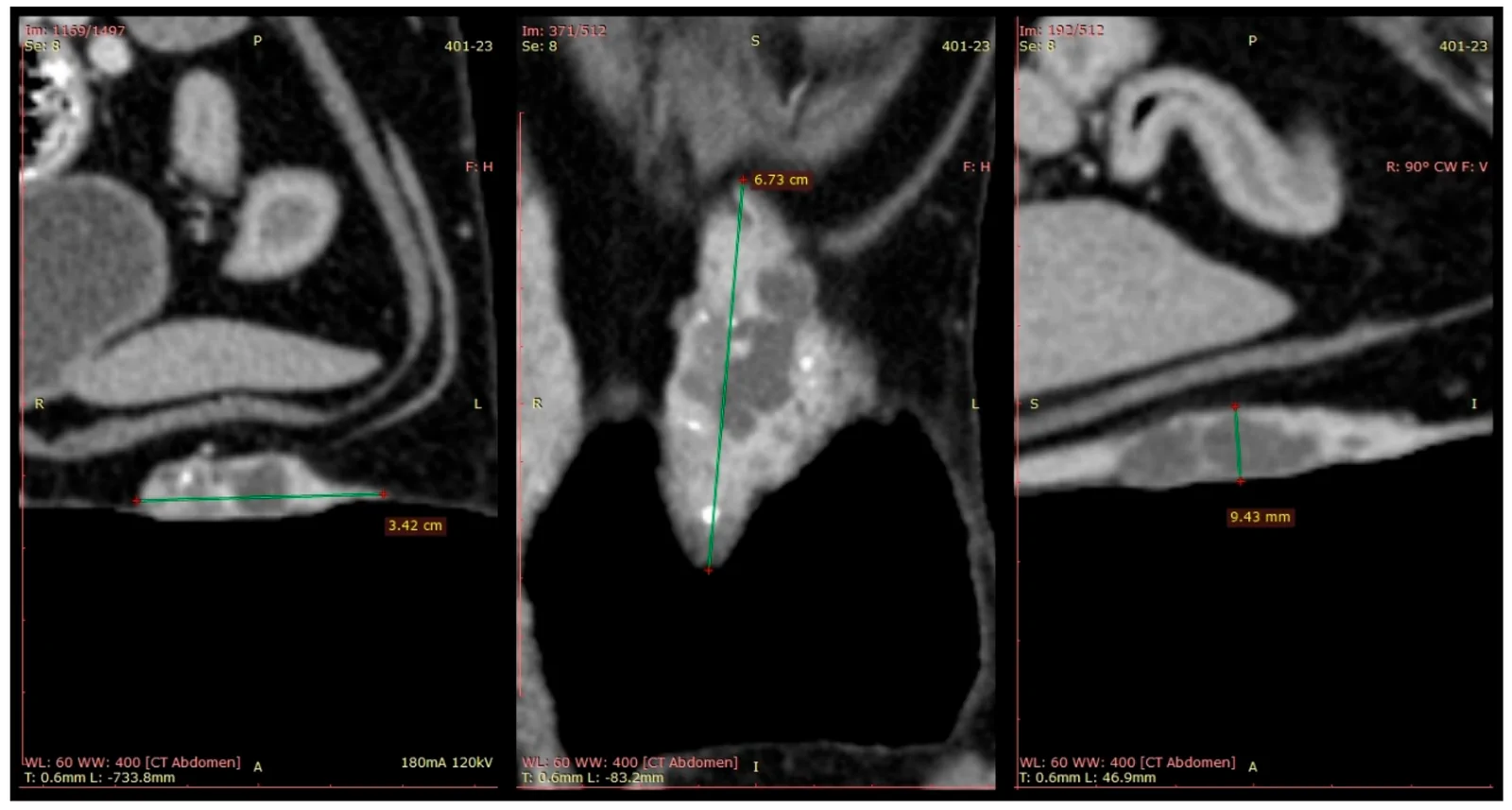
CT image of CMT of the left inguinal mammary gland (M5): measurement of height, width and length on a multiplanar reconstruction, followed by calculation of the nodular volume. The CMT shows perilesional oedema, nodular volume >1.9 cm^3^ and heterogeneous enhancement; it is assigned a score of 3 points, placing it in the high−risk category. Histological diagnosis: Mammary adenocarcinoma.

The studies were assessed in the transverse plane, and multiplanar reconstructions were used to optimise lesion visualisation. The tumours were evaluated in terms of size, shape, margins and parenchymal texture. The nodular size was assessed by measuring the maximum diameter in height (H), length (L) and width (W), and then calculating the volume using the formula for an ellipsoid—V = (4/3) × π * (H/2) × (L/2) ×(W/2)—where H, L and W represent the axes of the ellipsoid as the ventro-dorsal, cranio-caudal and latero-lateral projections, respectively (Figure 1). This is an easy and readily applicable measurement tool in routine radiological practice that does not require dedicated segmentation software.

**Figure 2:**
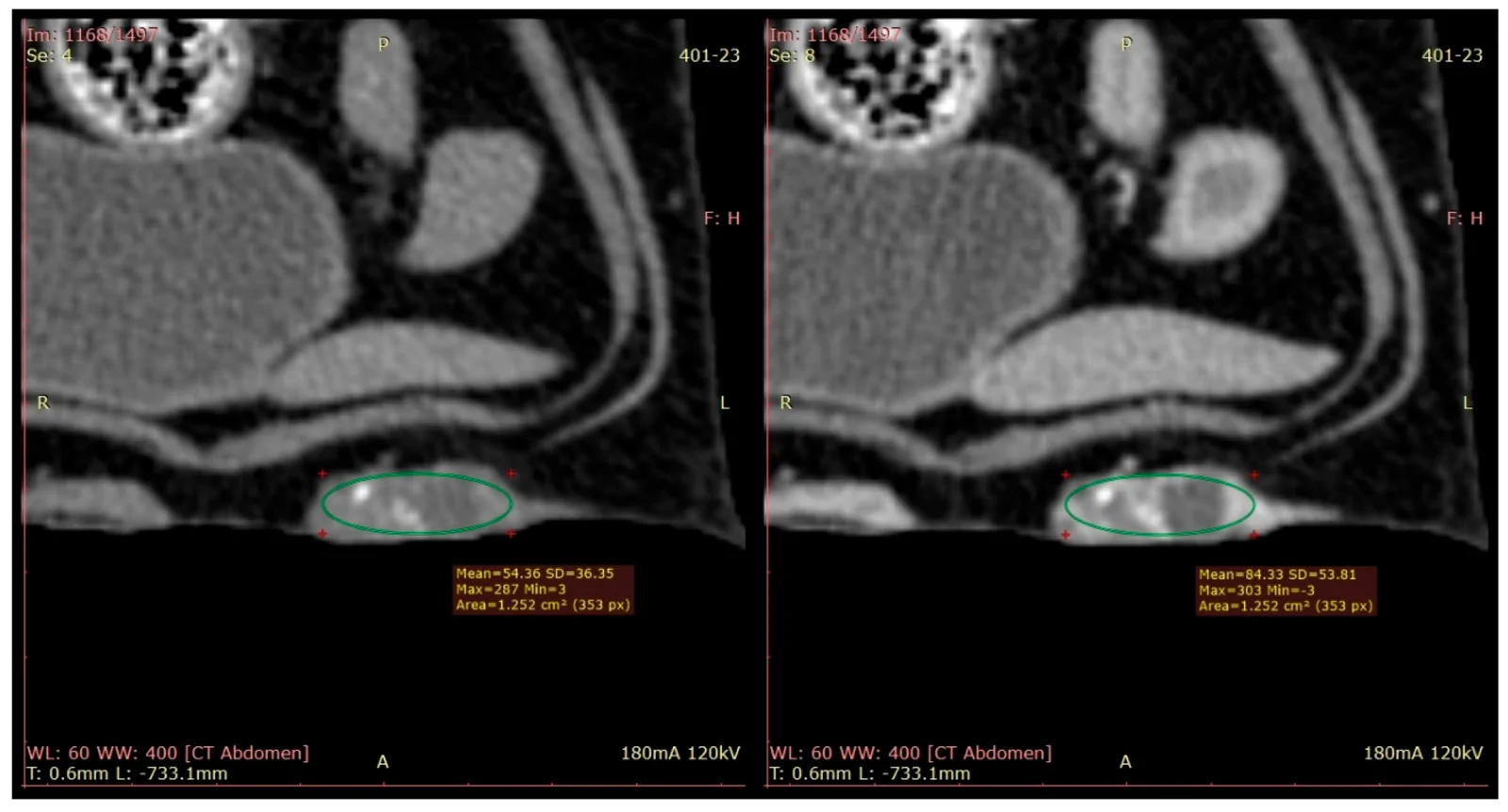
Transverse pre- and post-contrast CT images of left abdominal mammary gland (M4) with a single CMT: measurement of enhancement pre- and post-contrast administration, using a single ROI (green ellipse) in Radi-Ant DICOM Viewer (2025.2).

The quantitative analysis also included CT attenuation measurements (in Hounsfield units—HU) obtained on both pre- and post-contrast series using a single elliptical region of interest (ROI) positioned on the image slice displaying the largest tumour diameter and encompassing the maximum representative tumour area (Figure 2).

**Figure 3:**
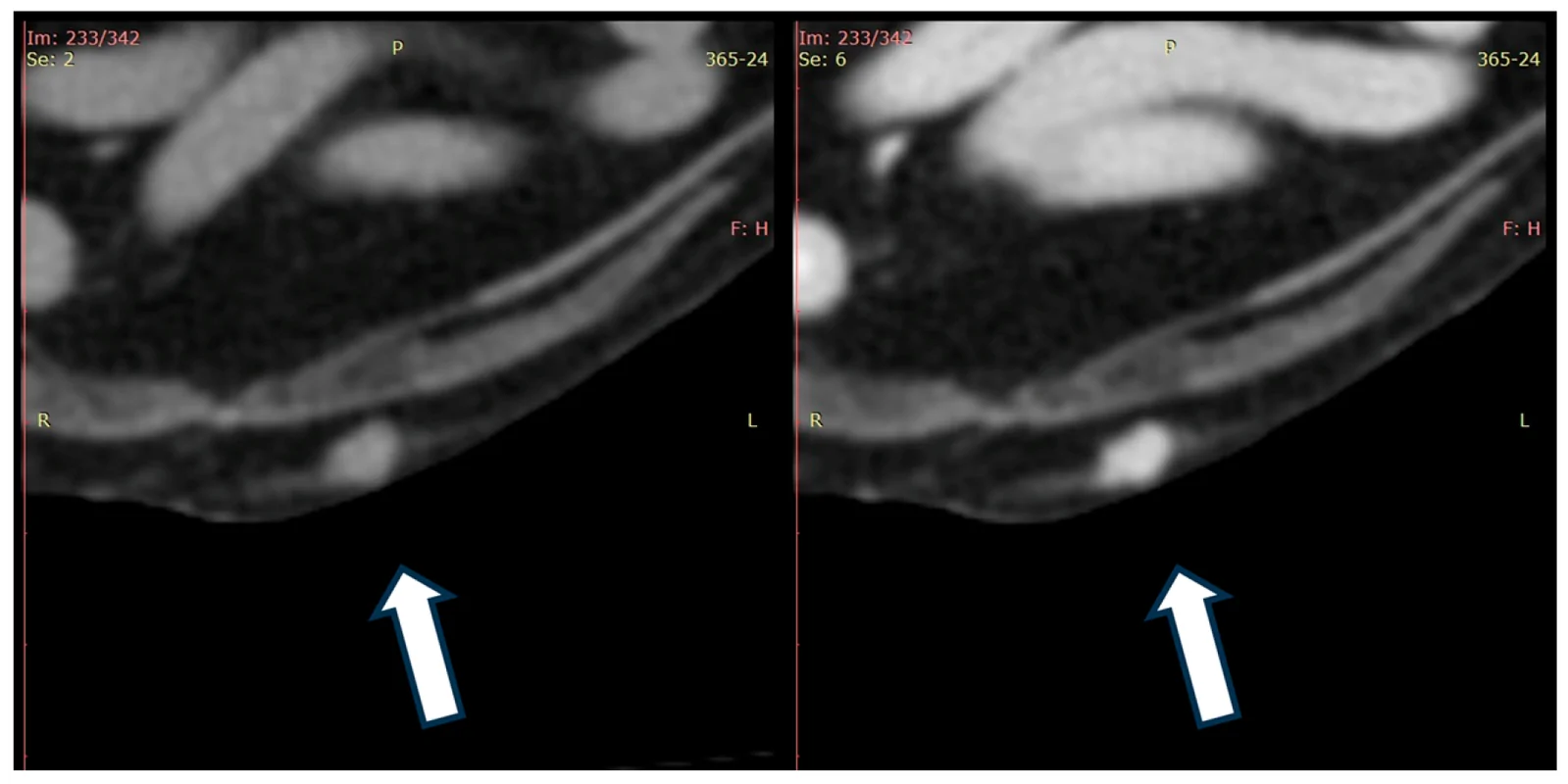
Transverse pre- and post-contrast CMT (white solid arrow) in left abdominal mammary gland (M4) presents with irregular shape, parallel orientation, circumscribed margins, homogenous enhancement pattern, no presence of calcification, perilesional oedema and vascularisation.

**Figure 4:**
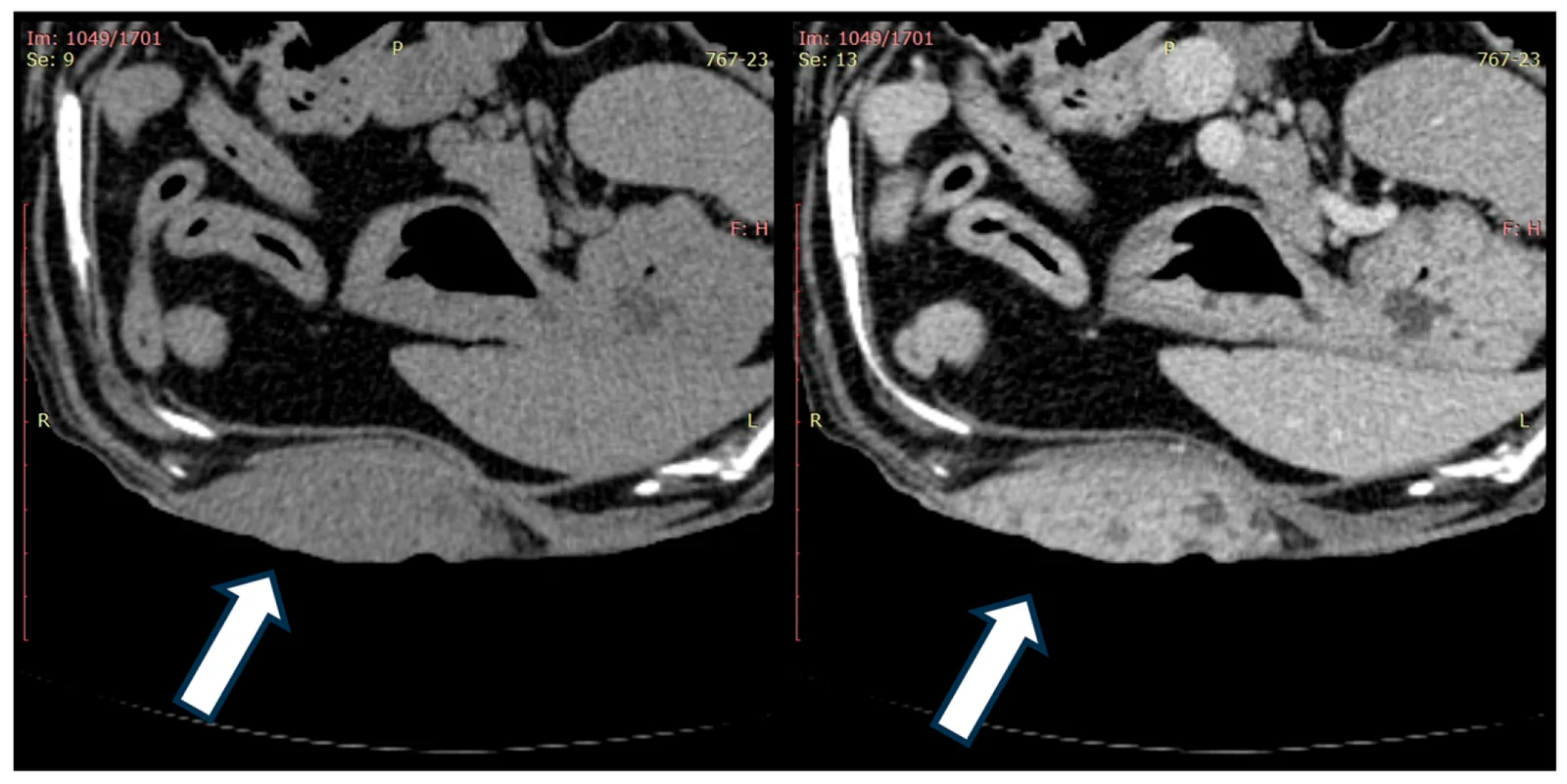
Transverse pre- and post-contrast CMT (white solid arrow) in right abdominal mammary gland (M3) with irregular shape, parallel orientation, not circumscribed margins, heterogeneous enhancement pattern, presence of vascularisation, without areas of calcifications and with discreet perilesional oedema.

**Figure 5:**
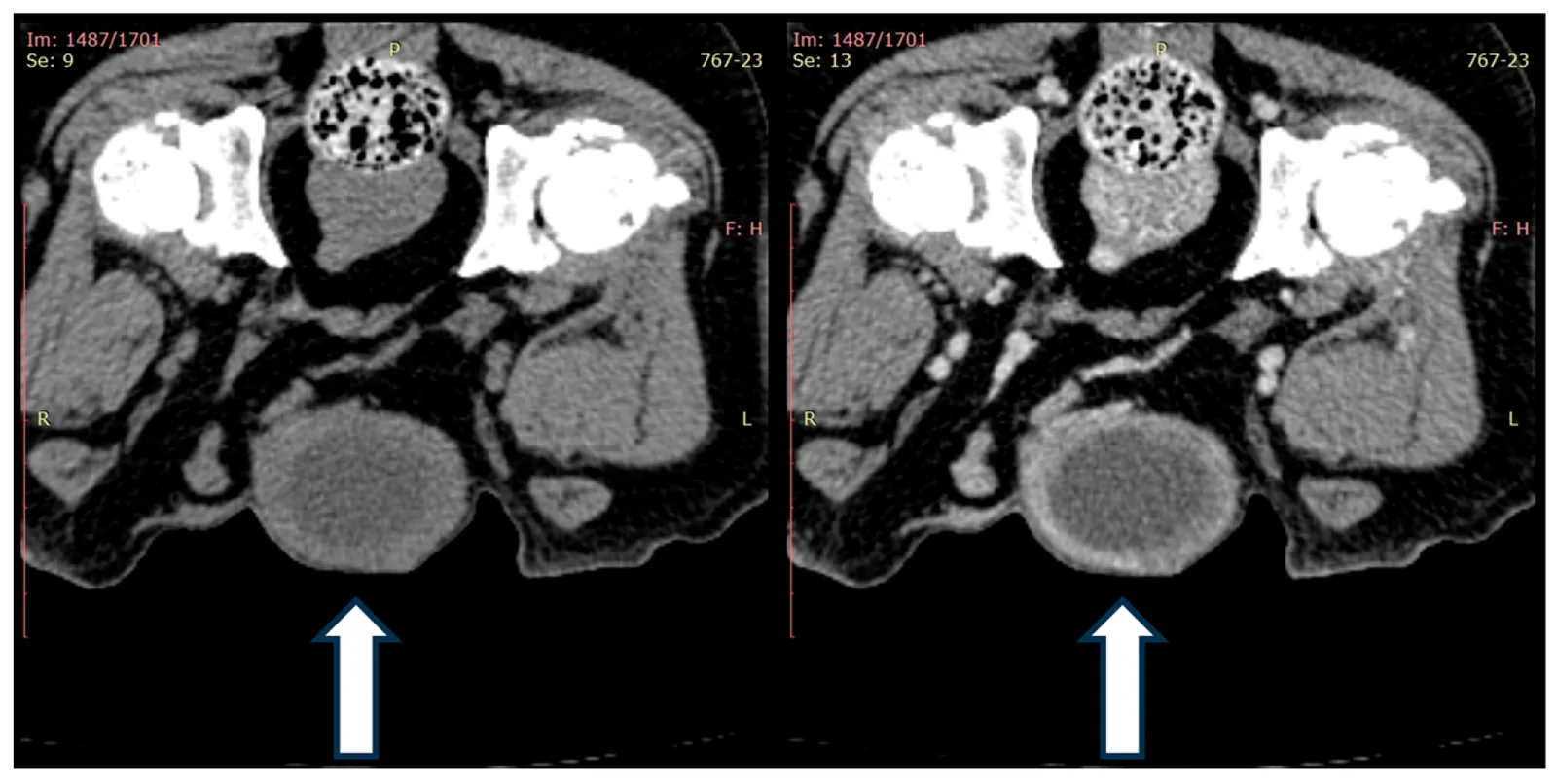
Transverse pre- and post-contrast CMT (white solid arrow) in right inguinal mammary gland (M5) with oval shape, parallel orientation, circumscribed margins, rim enhancement pattern, without presence of vascularisation and without areas of calcifications and perilesional oedema.

For the qualitative assessment of the CMTs and standardisation of the nomenclature across imaging modalities, the modified BI-RADS™ lexicon was adapted for CT lesion description (Table 1; Figure 3, Figure 4 and Figure 5). A lesion’s shape was classified as oval or irregular; its orientation as parallel or not parallel to the cutaneous surface; and its margins as circumscribed when considered well defined, or not circumscribed when indistinct. On the post-contrast CT images, enhancement was classified as homogeneous (uniform), heterogeneous (not uniform), or rim enhancement (predominantly peripheral). Calcifications were recorded according to their presence and location within or outside the mass. Perilesional oedema was assessed through increased attenuation and stranding of the surrounding fat, while vascularisation was recorded according to the presence or absence of identifiable contrast-enhancing vessels.

The enhancement pattern, presence of oedema and Volume Score were used to create a scoring system of 0–4 points (Figure 3). Homogenous, heterogeneous and rim enhancement were assigned 0, 1 and 2 points based on their progressively stronger association with malignancy; one additional point was assigned for presence of perilesional oedema; and one for tumour volume >1.9 cm^3^. Subsequently, two categories of risk were assigned: medium risk (<2 score) and high risk (score ≥2).

### 2.3. Statistical Analysis

All data were stored and processed using Microsoft Excel (Version 2606 Build 16.0.20131.20112). Statistical analyses were performed using SPSS (version 30.0). All quantitative data were assessed for normality by a Shapiro–Wilk test and the descriptive data were reported accordingly. Furthermore, the quantitative data were compared between the malignant and benign groups using a Mann–Whitney test, and where the results were statistically significant, ROC curves were used to assess the sensitivity and specificity. To evaluate the association between tumour volume and malignancy, the CMTs were stratified into two volume categories (Volume Score) based on the Receiver Operating Characteristic (ROC) analysis results and the optimal Youden’s index. The same procedure was applied to verify the association between an increase in maximum diameter and malignancy, using 1.4 cm (Max. Diameter) as the optimal cut-off value obtained from the statistical analysis, and obtaining two Max. Diameter categories. The qualitative data, including the Volume Score and Maximum Diameter, were compared between groups using Fisher’s exact test and a Chi-square test and, where statistical significance was found, the sensitivity and specificity were calculated.

A binary logistic regression was used to assess the combined and predictive effects of enhancement, the presence of oedema and the Volume Score within the model. Subsequently, the ROC curve and Youden’s index were used to determine the overall accuracy and the optimal cut-off for the score. A linear association test was applied to verify the progressive increase in malignancy as the score increased (*p* < 0.05).

To account for multiple comparisons, a Bonferroni correction was applied to the exploratory univariable comparisons that were initially considered significant. Both the unadjusted and Bonferroni-adjusted *p*-values were considered when interpreting the results.

To account for clustering between individual patients and mammary tumours, generalised estimating equations (GEEs) were subsequently used. A binomial distribution with a logit link function, an exchangeable working correlation structure and a robust co-variance estimator were specified, with the patient’s ID included as a subject variable. The enhancement pattern, perilesional oedema and tumour volume class (>1.90 cm^3^) were initially assessed as predictors in a cluster-adjusted multivariable model. The cumulative score of 0–4 points was subsequently assessed both as a continuous predictor and as a dichotomous risk classification (<2 vs. ≥2 points) (*p* < 0.05).

## 3. Results

Thirty-four female dogs, out of a total of sixty-one, were included, of which twenty-six were intact and eight neutered, with a median age of 10 years (IQR = 8–12), and with a median body weight of 8.2 kg (IQR = 2.5–39 kg). The dogs recruited represented various breeds, including Mix Breed (13), Maltese (six), Cocker Spaniel (two), Chihuahuas (two), Pinscher (two), Shih Tzu (one), Bedlington Terrier (one), Italian Greyhound (one), Labrador Retriever (one), West Highland Terrier (one), Dogo Argentino (one), German Shepherd (one), German Spitz (one), and Dachshund (one). A total of one hundred and six CMTs were identified in 34 dogs, with a median of 2.5 tumours per dog (IQR: 1–4; range: 1–15). The 106 CMTs were distributed across the five mammary glands as follows: M1 (*n* = 14; 13.2%), M2 (*n* = 4; 3.8%), M3 (*n* = 23; 21.7%), M4 (*n* = 37; 34.9%), and M5 (*n* = 28; 26.4%). Eighty-one tumours (76.41%) were histopathologically diagnosed as malignant, including 69 mammary carcinomas, nine adenocarcinomas, two tubulopapillary adenocarcinomas, and one carcinoma and malignant myoepithelioma.

In contrast, 25 tumours (23.59%) were classified as benign, comprising 22 adenomas and three benign mixed tumours. The distribution of tumour volumes showed marked positive skewness, with a median value of 2.30 cm^3^ (IQR = 0.79–9.54 cm^3^). The maximum diameter showed a stable distribution, with a median of 1.57 cm (IQR = 1.05–2.52 cm). No difference was observed between the groups concerning the parenchymal attenuation in the pre- and post-contrast series.

Among the qualitative features, only the enhancement pattern and the presence of oedema were different between the groups.

The remaining qualitative parameters, including the shape, margins, calcification, and vascularity, were not significantly different between the benign and malignant lesions; all 106 nodules presented the same parallel orientation, due to zero statistical variation between groups. As for the quantitative parameters, this difference was evident only for the height, volume and maximum diameter. A heterogeneous enhancement pattern and rim enhancement pattern were strongly associated with malignancy (*X*^2^ = 10.84; *p* < 0.001; 88.9% sensitivity, 40% specificity). As for oedema, it showed 33.3% sensitivity and 84% specificity for malignancy. Height was significantly associated with malignancy in the non-parametric tests and ROC curves (*p* = 0.038); however, it was not statistically significant in the linear regression models (OR = 1.15; 95% IC = 0.80–164; *p* = 0.456).

An increased nodular volume was significantly associated with higher chances of malignancy (AUC = 0.634; IC 95%: 0.507–0.761; *p* value = 0.04), showing high specificity (76%) but moderate sensitivity (50.6%). For this reason, the optimal cut-off value determined by the Youden index (J = 0.217) was established at 1.90 cm^3^ and was shown to be significantly associated with malignancy using Fisher’s exact test (*p* = 0.046), with a sensitivity of 61.7% and a specificity of 60%. In accordance with the observations regarding volume, an increase in the maximum nodule diameter was also significantly associated with a higher risk of malignancy when assessed as a continuous variable in the ROC curve analysis (AUC = 0.635; IC 95%: 0.510–0.759; *p* = 0.034). Application of the Youden index (J = 0.205) identified an optimal cut-off at 1.40 cm, which had a sensitivity of 60.5% and a specificity of 60%. However, the dichotomous classification of the variable based on this cut-off did not reach statistical significance in Pearson’s Chi-square test (*X^2^* = 3.251; *p* = 0.071).

To assess the combined predictive effect of the parameters, a multivariate binary logistic regression was initially performed, including the enhancement pattern, perilesional oedema and Volume Score. The overall model proved statistically significant (*X^2^* = 12.774; *p* = 0.012; Nagelkerke’s R^2^ = 0.171). Within this tumour-level model, the enhancement pattern emerged as the most robust independent predictor: rim enhancement was associated with a risk of malignancy approximately six times higher than the reference level (OR = 5.932; 95% CI= 1.523–23.099; *p* = 0.010). At the same time, the presence of oedema and a Volume Score showed positive risk trends (OR = 2.327 and OR = 1.515, respectively) (Table 2).

**Figure 6:**
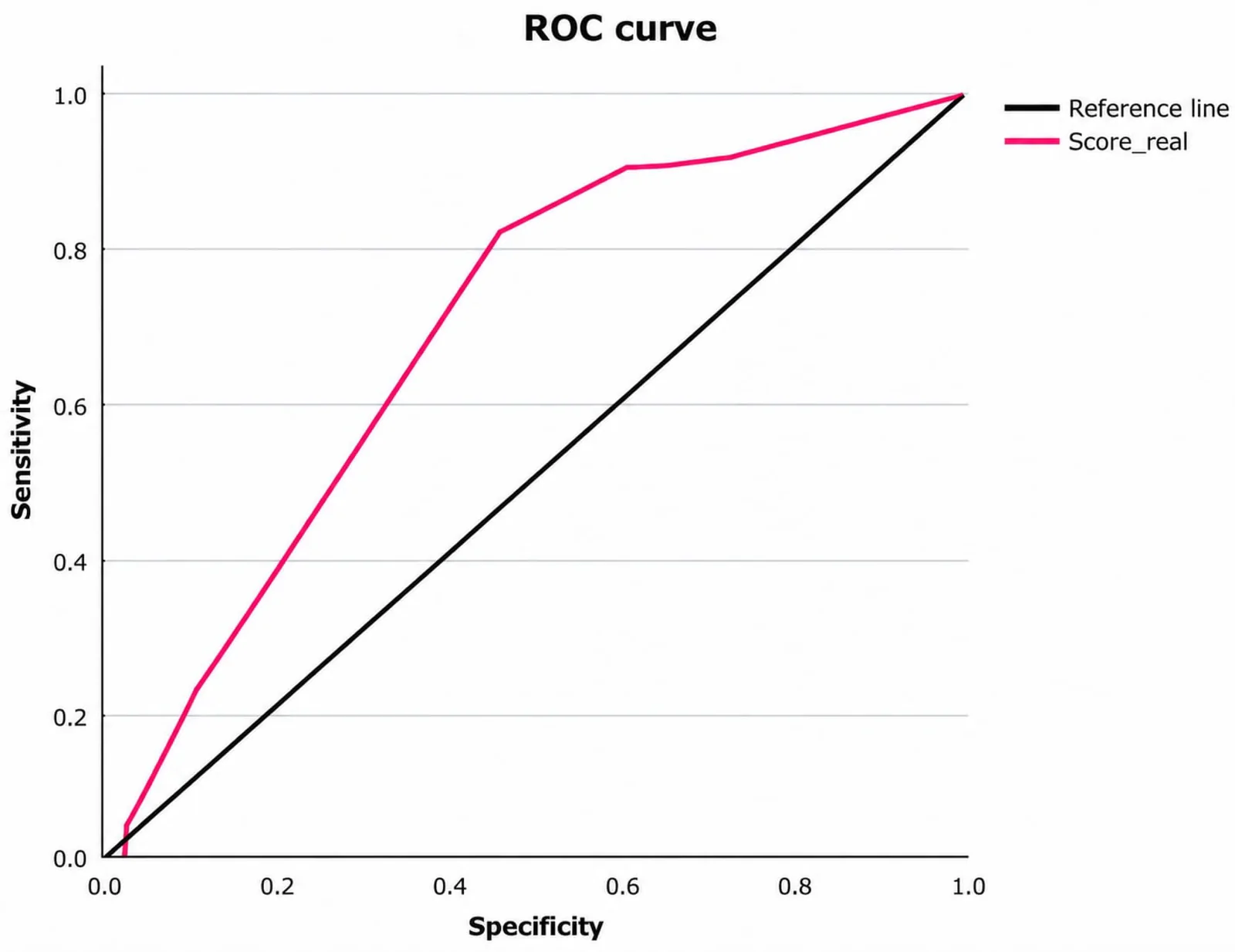
ROC curve for the cumulative CT scoring system for predicting malignancy in CMTs.

This predictive consistency justified the creation of a cumulative scoring system designed to capitalise on the additive and synergistic effects of the three variables. The scoring system consisted of the attribution of 1 point in presence of heterogeneous enhancement, 2 points in presence of rim enhancement, 1 point in presence of perilesional oedema, and 1 point when the tumour showed a volume ≥ 1.9 cm^3^. The scoring system, ranging from 0 to 4 points, demonstrated a statistical association with malignancy (AUC = 0.711; 95% CI = 0.590–0.831; *p* = 0.001) (Figure 6); applying Youden’s index (J = 0.389), two risk classes were identified: medium risk with a score <2 and high risk with a score ≥2, with a sensitivity of 82.7% and a moderate specificity of 58%.

This overall association was confirmed by a Chi-square test, and a significant trend was observed between the risk class and histological diagnosis (*X*^2^ = 15.234; *p* = 0.004). However, following a Bonferroni correction for multiple comparisons, only the association between enhancement pattern and malignancy was significant (*p* = 0.0159). The associations observed for tumour height, volume, and maximum diameter were not statistically significant after correction. Perilesional oedema and the tumour volume threshold of >1.90 cm^3^ were also not significantly associated with malignancy after adjustment.

Moreover, considering that many patients had multiple tumours, the scoring system was evaluated using a cluster-adjusted generalised estimating equation (GEE) model. The increase in a score from 0 to 4, as a continuous variable, showed a positive association but was not statistically significant (OR = 1.27; 95% CI = 0.95–1.71; *p* = 0.112). In contrast, when using the two risk categories (medium risk < 2 and high risk ≥ 2), the high-risk classification showed a statistically significant association with malignancy after cluster adjustment (OR = 1.70; 95% CI = 1.11–2.58; *p* = 0.014).

Considering the tumour-level model, a score of two or higher was associated with a significant increase in the probability of malignancy (84.2%), whilst scores below two were associated with malignancy in only 39.1% of cases, according to the linear association test (*p* < 0.001) (Table 3), suggesting that the two-class score may help identify high-risk malignant lesions (Figure 6).

## 4. Discussion

This study represents the first systematic evaluation of a newly standardised CT reporting protocol for CMTs, based on the adaptation of the BI-RADS™ lexicon, modified according to the criteria proposed for ultrasound by Oliveira et al. (2018) [20]. Although CT is widely validated for oncological clinical staging and the detection of pulmonary metastases [15,16], its ability to distinguish the nature of primary mammary nodules in dogs has remained poorly documented. Our results indicate that the integration of qualitative and quantitative parameters could potentially transform CT into a predictive tool of diagnostic significance pending further studies.

Among the parameters evaluated, the enhancement pattern proved to be the most predictive of malignancy. In particular, both heterogeneous and rim enhancement were found to be significantly more frequent in malignant lesions (overall sensitivity, 88.9%).

This finding can likely be explained by the neoplastic histopathological nature of malignant lesions, which are characterised by disorganised angiogenesis and therefore uneven distribution, associated with necrosis, haemorrhage, and tissue hypoperfusion, resulting in a heterogeneous and irregular distribution of the contrast agent within the lesion, which may therefore manifest as diffuse non-uniform enhancement or as peripheral enhancement (rim enhancement) [3]. Similarly, this finding has already been documented in human medicine, where recent studies on mammography and cone-beam breast CT have shown that heterogeneous and peripheral patterns are associated with a higher probability of malignancy, specifically, a strong association between rim enhancement and malignancy [24,25].

In essence, the data suggest that it is not the overall intensity of the contrast agent, but its distribution within the lesion that provides the most information regarding potential malignancy.

Another parameter frequently present in malignant tumours is the presence of oedema, which in our study showed high specificity (84%) but low sensitivity (33.3%). This finding suggests that, although oedema is not frequently detectable and by itself it is statistically inconclusive, its presence, in association with other specific patterns, can provide a diagnostic clue and may be attributed to alterations in the stromal microenvironment resulting from neoplastic infiltration, compression or obstruction of lymphatic drainage, and the associated inflammatory response [26].

Similarly, tumour volume is confirmed as a parameter associated with the risk of malignancy, showing high specificity (76%) but moderate sensitivity (50.6%). However, in a clinical context, giving absolute priority to mathematical specificity carries a serious risk of generating false negatives, with the possibility of missing early-stage or low-volume malignant tumours. This critical limitation is strongly supported by Burrai et al. (2020) [7], who demonstrated that up to 18% of malignant lesions may have a diameter of less than 1 cm. In order to minimise the risk of false negatives, we deliberately selected a clinically optimised cut-off of 1.90 cm^3^, in accordance with Youden’s index. This optimal cut-off provided a more reliable and balanced diagnostic profile, raising the sensitivity to 61.7% (with a specificity of 60.0%). Once dichotomised, this volume threshold showed a positive risk trend in the multivariate logistic regression (OR = 1.515), but after the Bonferroni correction, the adjusted *p* value was not significant. This highlights, as demonstrated for oedema, that as individual continuous parameters, they do not have a strong discriminatory ability for malignancy, but they achieve statistical significance when considered as a system. Nevertheless, it is important to consider that the ellipsoid volume formula may provide less accurate volume estimates for irregularly shaped tumours compared with manual or semi-automated segmentation, despite being a practical and easily performed measurement. Although a direct comparison between these methods was not performed, future studies should investigate the accuracy of ellipsoid-based volume estimation, particularly in lesions with irregular morphology.

In this study, the presence of calcifications surprisingly did not prove to be a discriminating factor for malignancy in our sample, contrary to the findings of Kim et al. (2017) [21], who instead attributed moderate sensitivity to this parameter as a predictor of malignancy. Therefore, while Kim et al. evaluated the calcification morphology, our analysis focused on their presence and location. This apparent discrepancy may be associated with the unique histological heterogeneity of canine mammary tumours. In fact, CMTs frequently exhibit mixed epithelial and mesenchymal components, with documented instances of bone and cartilage metaplasia [23,27], in addition to possible secondary processes, such as necrosis, fibrosis, or inflammatory sequelae. However, this interpretation remains controversial; further studies incorporating calcification morphology and distribution are therefore warranted.

The scoring system proposed within the present study synthesises this multivariable approach by integrating enhancement patterns, the presence of oedema, and tumour volume. Despite this approach showing moderate discriminatory power (AUC = 0.711) at the tumour level, after applying a Bonferroni correction for multiple comparisons, the association between the dichotomised enhancement pattern and malignancy was statistically significant, whereas the association between a volume >1.9 cm^3^ and malignancy was not. The exploratory nature of the analyses and the limited sample size should be taken into consideration when interpreting these results. Further studies involving larger populations are necessary to confirm these associations and validate the proposed multiparametric scoring system. Nevertheless, it demonstrated moderate significance at identifying high-risk lesions (>2 points) following cluster adjustment (OR 1.70; *p* < 0.014). Furthermore, the rather small number of benign tumours and the number of predictors included in the multivariable model may have increased the risk of overfitting. Therefore, the proposed scoring system should be considered preliminary, and further validation in larger and independent multicentre populations is warranted.

This study has limitations that should be considered. Although the study’s inclusion criteria selected only CT scans performed using rigorously standardised acquisition protocols, thus ensuring a high degree of homogeneity in the sample, the multicentre and retrospective nature of the study inevitably involves the use of CT scanners with different technical characteristics (e.g., varying numbers of slices and different reconstruction algorithms). This purely technological variability could have influenced the spatial resolution of minor morphological details and the absolute quantification of attenuation values (HU).

Another limitation related to the retrospective nature of the study is the use of different anaesthetic protocols, which could have influenced the cardiovascular dynamics and consequently the contrast medium distribution. Therefore, residual confounding factors cannot be excluded, as these effects were not accounted for in the statistical analyses [28].

Furthermore, the low number of benign tumours, probably due in part to the use of RX rather than CT scans for the staging of small tumours, and consequently the predominance of malignant lesions in the analysed sample, may have overestimated the positive predictive value of certain parameters included in the scoring system. Nevertheless, some epidemiological studies have already demonstrated that the distribution between benign and malignant tumours is not uniform worldwide, and that the prevalence of malignancy can be higher than benign lesions [3,14]. In Italy, a study demonstrated that the prevalence of CMTs was 70%, while benign cases represented 30% [10], which is somewhat similar to the findings of the present study, which was conducted in the same country. Therefore, although the distribution of malignant and benign tumours in this study was not uniform and might have introduced statistical bias, it might accurately represent the incidence of malignancy in this country.

The present study was primarily designed to develop a simple, rapid, and clinically applicable CT analysis and reporting protocol for the characterisation of primary mammary lesions. Accordingly, the proposed scoring system was based exclusively on routinely assessable CT features, without requiring histopathological grading for its application. Similarly, regional lymph node involvement and distant metastases were not analysed in relation to the imaging characteristics of primary tumours, which are important prognostic factors. Further investigations are warranted to assess the relationships among the proposed CT features, histological grade, lymph node status, and distant metastases to assess their potential prognostic relevance.

Future studies should focus on assessing the reproducibility of the results and inter-observer agreement, with the aim of verifying consistency in the application of this adapted BI-RADS™ lexicon amongst radiologists with varying levels of clinical experience. Additionally, future studies should focus on expanding the multicentre approach, involving veterinary institutions from different geographical regions, which could potentially provide a more balanced distribution of benign and malignant tumours.

## 5. Conclusions

This study demonstrates the feasibility of a modified BI-RADS™ lexicon adapted for CT for characterising canine mammary tumours, which can be used to optimise radiological reporting. Specific qualitative CT features, such as heterogeneous enhancement, rim enhancement, and presence of oedema, were significantly more associated with malignant nodules. The mammary tumour scoring system based on medium- and high-risk classes showed preliminary discriminatory potential (AUC = 0.711) to evaluate the probability of malignancy in higher-risk categories, although its specificity was limited (58%).

Furthermore, this scoring system is a simple, intuitive and easy-to-use tool that can be further explored in controlled research or clinical environments without the need for complex external software. Nevertheless, given its low specificity, it should not be used as a standalone diagnostic tool, but rather as a complementary assessment within a comprehensive clinical evaluation.

Further prospective studies are warranted to validate these findings and refine this standardised CT reporting protocol for broader clinical use.

## Figures and Tables

**Table 1:** Modified BI-RADS™ lexicon for the evaluation of canine mammary tumours. The present lexicon is adapted from Harish et al., 2007 [22]; Mohammed et al., 2011 [23]; ACR BI-RADS™ (2013) [19]; Oliveira et al., 2018 [20]; and Feliciano et al., 2023 [17,18].

Features	Terminology
Shape	Oval
	Irregular
Orientation	Parallel
	Not parallel
Margins	Circumscribed
	Not circumscribed
Enhancement Pattern	Homogeneous
	Heterogeneous
	Rim enhancement
Presence of Calcification	Inner-mass calcification
	Outer-mass calcification
	Absent
Perilesional Oedema	Present
	Absent
Vascularisation	Present
	Absent

**Table 2:** Diagnostic performance of individual CT parameters for predicting malignancy in CMTs.

CT Parameter	Metric/Sensitivity (%)	Specificity (%)	ROC (AUC) 95% IC	Test Statistic (*X^2^*/U)	Odd Ratio 95% IC	Statistical Significance	Adjusted *p*-Value (Bonferroni Correction)
Statistically Significant Parameters (*p* < 0.05)							
Enhancement pattern (homogeneous vs. heterogeneous/rim)	88.9%	40.0%	0.689 (0.57–0.81)	X^2^ = 10.84, df = 2	5.93 (1.52–23.10)	0.001	0.015
Height (continuous, cm)	-	-	0.638 (0.51–0.77)	U = 733.5, Z = −2.08	1.15 (0.80–1.64)	0.038	
Nodular volume (continuous, cm^3^)	-	-	0.634 (0.51–0.761)	U = 741.0, Z = −2.02	-	0.04	0.640
Maximum diameter (continuous, cm)	-	-	0.635 (0.510–0.759	U = 739.5, Z = −2.03	-	0.042	0.672
Nodular volume (>1.90 cm^3^)	63%	60%	-		2.42 (0.97–6.03)	0.046	0.736
Statistical Trends (0.05 ≤ *p* < 0.10)							
Perilesional oedema	33.3%	84%	-	X^2^ = 2.77, df = 1	2.63 (0.82–8.41)	0.096	1
Maximum diameter (>1.40 cm)	60.5%	64.0%	-	X^2^ = 3.24, df = 1	2.30 (0.92–5.72)	0.071	
Width (continuous, cm)	-	-	0.610 (0.48–0.74	U= 790.0, Z= −1.66	**-**	0.098	
Statistically Non-Significant Parameters (*p* ≥ 0.10)							
Post-contrast attenuation (HU, continuous)	-	-	0.589 (0.460–0.720)	U= 837.5, Z= −1.30	-	0.193	
Pre-contrast attenuation (HU, continuous)	-	-	0.513 (0.380–0.650)	U = 992.5, Z = −0.15	-	0.882	
Percentage enhancement (%, continuous)	-	-	0.548 (0.420–0.680)	U = 921.0, Z = −0.68	-	0.496	
Length (continuous, cm)	-	-	0.580 (0.450–0.710)	U = 857.0, Z = 1.16	-	0.247	
Shape (irregular vs. oval)	29.6%	80.0%	-	X^2^ = 0.89, df = 1	1.68 (0.56–5.00)	0.345	
Margins (circumscribed vs. not circumscribed)	30.9%	76%	-	X^2^ = 0.44, df = 1	1.41 (0.50–3.95)	0.510	
Vascularity (present vs. absent)	19.8%	72%	-	X^2^ = 0.77, df = 1	1.58 (0.56–4.41)	0.382	
Calcification (present vs. absent)	27.2%	84.0%	-	X^2^ = 1.29, df = 1	1.96 (0.60–6.33)	0.257	

**Table 3:** Results of CT mammary scoring system and risk assessment. This table integrates the point-based scoring criteria, the cumulative diagnostic performance at tumour level and after the cluster-adjustment for a single patient, and the associated risk of malignancy.

Diagnostic Metrics for Score > 2	Terminology
Score range	0–4
ROC AUC	0.711
High-risk threshold	≥2
Sensitivity	82.7%
Specificity	58%
Cluster-adjusted OR, score ≥2 vs. <2	1.70
Linear association test	*p* < 0.001
GEE *p*-value	0.014

## Data Availability

The data supporting the findings of this study are available from the corresponding author upon reasonable request. The data are not publicly available due to privacy and confidentiality considerations related to clinical records of client-owned animals.

## Abbreviations

The following abbreviations are used in this manuscript:

CMTs	Canine Mammary Tumours
CT	Computed Tomography
WBCT	Whole-Body Computed Tomography
XR	Radiography
US	Ultrasonography
BI-RADS	Breast Imaging Reporting and Data System
ROI	Region of Interest
HU	Hounsfield Units
WL	Window Level
WW	Window Width
CI	Confidence Interval
IQR	Interquartile Range
GEE	Generalised Estimating Equations
TNM	Tumour–Node–Metastasis
